# A Competitive High‐Throughput Screening Platform for Designing Polylactic Acid‐Specific Binding Peptides

**DOI:** 10.1002/advs.202303195

**Published:** 2023-08-23

**Authors:** Yi Lu, Kai‐Wolfgang Hintzen, Tetiana Kurkina, Yu Ji, Ulrich Schwaneberg

**Affiliations:** ^1^ Institute of Biotechnology RWTH Aachen University 52074 Aachen Germany; ^2^ DWI‐Leibniz Institute for Interactive Materials 52074 Aachen Germany

**Keywords:** binding specificity, directed evolution, high‐throughput screening, material binding peptides, mixed plastics, polylactic acid, polypropylene

## Abstract

Among biobased polymers, polylactic acid (PLA) is recognized as one of the most promising bioplastics to replace petrochemical‐based polymers. PLA is typically blended with other polymers such as polypropylene (PP) for improved melt processability, thermal stability, and stiffness. A technical challenge in recycling of PLA/PP blends is the sorting/separation of PLA from PP. Material binding peptides (MBPs) can bind to various materials. Engineered MBPs that can bind in a material‐specific manner have a high potential for material‐specific detection or enhanced degradation of PLA in mixed PLA/PP plastics. To obtain a material‐specific MBP for PLA binding (termed PLA^bodies^), protein engineering of MBP Cg‐Def for improved PLA binding specificity is reported in this work. In detail, a 96‐well microtiter plate based high‐throughput screening system for PLA specific binding (PLABS) was developed and validated in a protein engineering (KnowVolution) campaign. Finally, the Cg‐Def variant V2 (Cg‐Def S19K/K10L/N13H) with a 2.3‐fold improved PLA binding specificity compared to PP was obtained. Contact angle and surface plasmon resonance measurements confirmed improved material‐specific binding of V2 to PLA (1.30‐fold improved PLA surface coverage). The established PLABS screening platform represents a general methodology for designing PLA^bodies^ for applications in detection, sorting, and material‐specific degradation of PLA in mixed plastics.

## Graphical abstract

A PLA binding specificity screening system based on PLA coated 96‐well MTPs for material binding peptide engineering is established and validated by improving PLA binding specificity over PP through a full KnowVolution campaign. The generated MBP variant exhibits enhanced PLA binding specificity over PP and has potential applications in a circular polymer economy through material‐specific labeling of polymer materials and targeted depolymerization of mixed plastic waste.

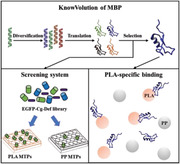

## 1 Introduction

Polylactic acid (PLA) is one of the most promising and rapidly‐growing bioplastics (growth rate of 39% by 2027) among biobased polymers with an expected production of more than 300 000 tons in 2024.^[^ ^1^, ^2^ ^]^ PLA is used in various applications such as food packaging, agricultural films, and drug delivery.^[^ ^3^, ^4^ ^]^ PLA is often blended with polypropylene (PP) in order to match performance demands in applications, such as melt processability, thermal stability, and stiffness.^[^ ^5^, ^6^, ^7^ ^]^ An increase in PLA production will require the development of new recycling processes in the future, in which PLA can be sorted from other polymers (e.g., PP and polyethylene terephthalate (PET)) in the waste.^[^ ^8^ ^]^

Material binding peptides (MBPs) have outstanding binding properties to a variety of materials including synthetic polymers (e.g., PP,^[^ ^9^, ^10^ ^]^ polystyrene (PS),^[^ ^11^ ^]^ polyurethane,^[^ ^12^ ^]^ PET^[^ ^13^ ^]^). Furthermore, the binding properties of MBPs in presence of detergents and rain have been improved.^[^ ^13^, ^14^, ^15^, ^16^, ^17^ ^]^ MBPs were conjugated to Alexa‐fluorophores to label and sort microparticles particles by flow cytometry.^[^ ^18^ ^]^ Inspired by cellulases in nature, MBPs have been used as artificial binding domains to enhance depolymerization of polyester‐polyurethane particles,^[^ ^19^ ^]^ PET,^[^ ^20^ ^]^ and polyhydroxyalkanoate (PHA).^[^ ^21^ ^]^

Knowledge‐gaining directed evolution (KnowVolution) is a most universal protein engineering strategy since it is not limited to specific properties and extensive dataset.^[^ ^22^ ^]^ A KnowVolution campaign comprises four phases, in which computational design and experimental identification are combined to minimize experimental efforts, maximize improvements of desired properties in a short time, and thereby to generate a comprehensive molecular understanding of each beneficial substitution.^[^ ^9^ ^]^ KnowVolution has been applied to improve various protein properties including activity,^[^ ^23^ ^]^ thermal resistance,^[^ ^24^ ^]^ pH resistance,^[^ ^25^ ^]^ regioselectivity,^[^ ^26^, ^27^ ^]^ ionic liquid resistance,^[^ ^28^ ^]^ and polymer processivity.^[^ ^29^ ^]^ In addition, KnowVolution is employed to enhanced MBPs binding properties to different kinds of polymers.^[^ ^9^, ^11^, ^22^ ^]^ For example, KnowVolution was applied to engineer MBP Tachystatin A2 (TA2) employing the casting error‐prone polymerase chain reaction method (cepPCR) in Phase I. The generated TA2 variant TA2‐M1‐PS (R3S/L6P/V12 K/S15P/C29R/R30L/F33S//Y44H) showed a 6.3‐fold stronger PS binding compared to TA2 wild type (WT).^[^ ^11^ ^]^ Based on the latter report, we decided to explore the application of the KnowVolution in MBPs design for improved PLA binding specificity.

An essential for a successful directed evolution campaign is to have a reliable and robust screening system.^[^ ^11^ ^]^ Commercial microtiter plates (MTPs) are mainly made of PP or PS and a first 96‐well MTP‐based assay termed ABBA was reported to improve the PP and PS binding strength of the MBPs LCI and TA2.^[^ ^11^ ^]^ As expected, the coefficients of variation (14.2% for EGFP‐LCI WT on PP and 10.6% for EGFP‐TA2 WT on PS) were sufficiently low to prove reliability and robustness of established ABBA screening system. To our best knowledge, there is no report regarding a screening platform for designing PLA‐specific binding peptides (PLA^bodies^).

In this work, we reported the first PLA Binding Specificity (PLABS) screening system based on PLA coated 96‐well MTPs for PLA^bodies^ engineering. MBP Cg‐Def was selected due to its PLA and PP binding properties. The PLABS screening platform was validated by improving PLA binding specificity over PP through a full KnowVolution campaign. Competitive binding tests in one pot, surface plasmon resonance (SPR), and contact angle measurements confirmed improved binding ratios of PLA to PP by an engineered Cg‐Def variant.

## 2 Results and Discussion

**Figure 1:**
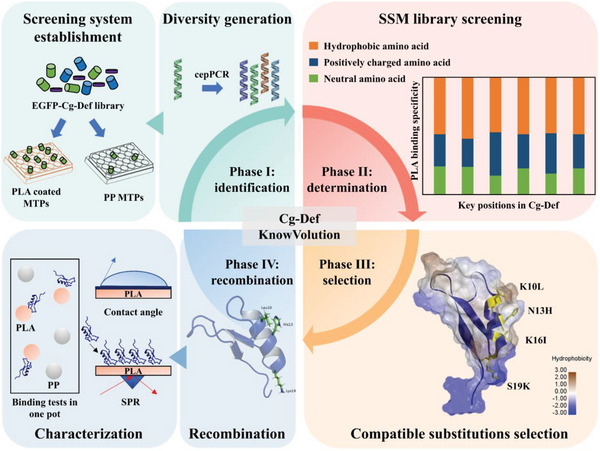
Enhanced PLA‐specific binding of Cg‐Def by KnowVolution. KnowVolution campaign comprising four phases was conducted to engineer Cg‐Def. In Phase I, cepPCR library of Cg‐Def with high mutation rate was generated. A high‐throughput screening platform based on PLA coated MTPs and PP MTPs for designing PLA‐binding peptides was established. After screening cepPCR library, potentially beneficial positions were identified. Then in Phase II, beneficial positions were determined after screening the SSM libraries of identified positions. In Phase III, the computational analysis of determined beneficial positions and substitutions was performed to find compatible substitutions. Finally, recombined variant was generated and characterized for PLA binding specificity by competitive binding tests in one pot, contact angle, and SPR measurements in Phase IV. PLA: polylactic acid; MBP: material binding peptides; PP: polypropylene; cepPCR: casting error‐prone polymerase chain reaction method; SPR: surface plasmon resonance; SSM: site‐saturation mutagenesis.

Cg‐Def variants with improved PLA binding specificity are of high importance to enable recycling processes, in which different mixed‐polymer waste has to be sorted (e.g., sorting PLA from PP) for a material‐specific recycling. The work described here was performed in four steps (**Figure** **1**). First, Cg‐Def was selected among 3 MBPs due to its PLA binding specificity. Second, PLABS screening system toward PLA specific binding compared to PP was established including a coating procedure for PLA on MTPs. Third, KnowVolution campaign comprising four phases was conducted to validate the screening system by yielding a Cg‐Def variant with improved PLA binding specificity. Fourth, obtained Cg‐Def variant with enhanced PLA binding specificity was characterized (competitive binding tests in one pot, contact angle, and SPR).

### 2.1 Selection of MBP with Potential PLA Binding Specificity

**Figure 2:**
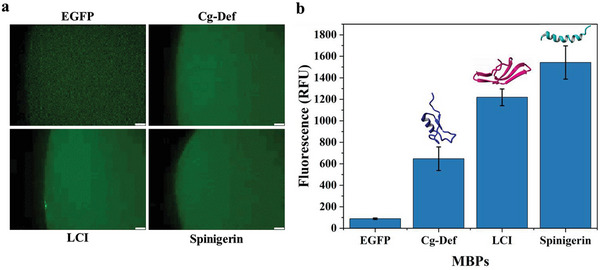
a) Fluorescence microscopy images of PLA granules bound with EGFP (negative control) and EGFP fusion proteins with Cg‐Def, LCI, and Spinigerin (scale bar represented 20 µm). b) Binding performance of EGFP, EGFP fusion proteins with Cg‐Def, LCI, and Spinigerin to PP. Experiments were performed in triplicate. The concentration of MBPs was 2.5 µM. The PLA granules and PP MTPs were washed two times with Tris‐HCl and one time with alkyl benzene sulfonate (LAS) after binding with MBPs (ambient temperature, pH of 8.0, 10 min).

The binding performances of three MBPs Cg‐Def, LCI, and Spinigerin (after purification) toward PLA and PP were evaluated by fluorescence measurements (fluorescence microscopy and fluorescence MTP reader). The binding of Cg‐Def, LCI, and Spinigerin on PLA were characterized by fluorescence images (**Figure** **2**a) and quantified fluorescence intensity (Figure S1, Supporting Information). The fluorescence values for Cg‐Def (82.6 A.U.), LCI (80.6 A.U.), and Spinigerin (55.5 A.U.) on PLA correlate well with the fluorescence images. Additionally, the lower fluorescence of Cg‐Def (647.3 RFU) on PP than LCI (1218.7 RFU) and Spinigerin (1542.3 RFU) was observed (Figure 2b). To explore the mechanism underlying PLA‐specific binding preferences of MBPs, PLA binding of Cg‐Def, LCI, and Spinigerin was analyzed in all‐atomistic molecular dynamics (MD) simulations using the GROMACS software.^[^ ^30^, ^31^, ^32^ ^]^ Binding was observed for all MBPs to PLA (N = 27). The gmx_MMPBSA tool was used to calculate the free‐energy of binding following the molecular mechanics/poisson‐boltzman surface area (MM/PBSA) approach.^[^ ^33^ ^]^ Binding energy analysis revealed that Cg‐Def exhibits the lowest binding energy on PLA among the three tested MBPs (Figure S2, Supporting Information), indicating the higher binding of Cg‐Def to the PLA surface than LCI and Spinigerin.

Thus, Cg‐Def was due to its material‐specific binding preference selected as MBP for PLABS system validation in a KnowVolution campaign with the goal to improve the PLA binding specificity over PP.

### 2.2 Establishing a Screening System for PLA Binding Specificity over PP

PLA MTPs are commercially not available. Therefore, a simple and robust coating procedure to coat PLA on PP MTPs was developed by dissolving PLA in dichloromethane. The optimized loading volume and concentration of PLA solution were 50 µL and 34 mg mL^−1^ (Figure S3a, Supporting Information). The developed procedure can be used for different polymers including PHA,^[^ ^34^ ^]^ PS,^[^ ^35^ ^]^ and acrylonitrile butadiene styrene (ABS)^[^ ^35^ ^]^ that could be dissolved in dichloromethane to coat PP based MTPs.

**Figure 3:**
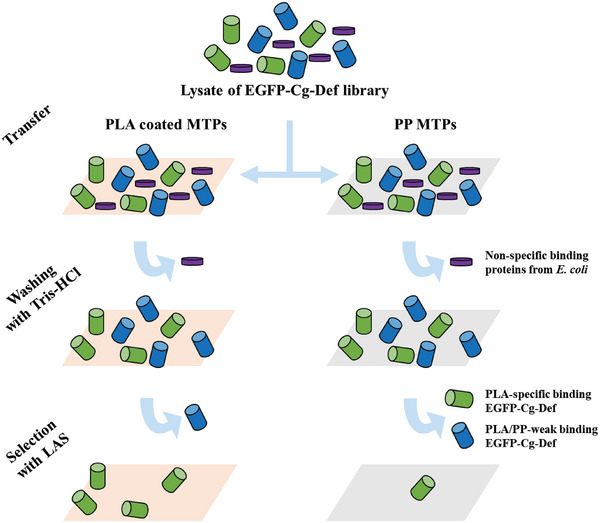
Overview of the PLABS system to identify Cg‐Def variants with improved binding PLA preference over PP by comparing the remaining fluorescence in PLA coated MTPs over PP MTPs after washing with Tris‐HCl buffer and LAS.

**Figure** **3** summarized the selection procedure used in the PLABS screening platform to improve the material‐specific binding of EGFP‐Cg‐Def variants to PLA over PP. Binding preference is determined through fluorescence measurements. In detail, lysates of EGFP‐Cg‐Def variants are transferred into PLA coated and PP MTPs for Cg‐Def mediated immobilization. Washing and purification of immobilized fusion proteins is performed with Tris‐HCl buffer. Surfactant alkyl benzene sulfonate (LAS) is later applied as selection pressure to remove weak binding EGFP‐Cg‐Def variants. Comparison of the remaining fluorescence in the PLA coated and PP MTPs is used as selection ratio for improved PLA‐specific binding.

In addition, to saturate the binding in PLA coated MTPs, 20 µL of EGFP‐Cg‐Def cell lysate was applied in screening system (Figure S3b, Supporting Information). Based on the optimized parameters, a coefficient of variation of screening system, which reflects the reliability of screening system in directed evolution campaign, was determined. As shown in Figure S4 (Supporting Information), the coefficient of variation of 18.1% was obtained for PLA and 19.1% was achieved for PP. Screening systems with a coefficient of variation of around 20% were successfully be used in directed evolution campaigns by rescreening of the most promising variants in multiple plates.^[^ ^36^, ^37^ ^]^

### 2.3 KnowVolution of Cg‐Def for Improving PLA Binding Specificity

**Figure 4:**
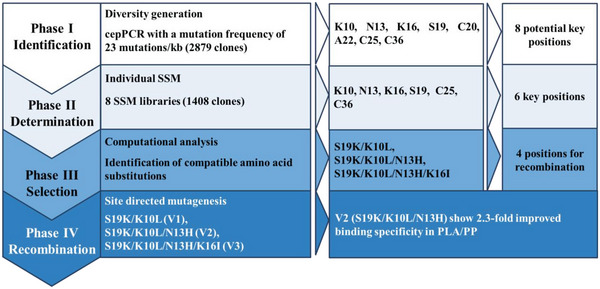
Overview of KnowVolution campaign of Cg‐Def for improving its PLA binding preference over PP. Phase I: a random mutagenesis library (cepPCR library) was generated and screened. 8 potentially beneficial positions (K10, N13, K16, S19, C20, A22, C25, and C36) were yielded after sequencing. Phase II: each position was subject to SSM using the wild type Cg‐Def as template and two 96‐well MTPs were screened per position. After screening the 8 SSM libraries, 6 positions (K10, N13, K16, S19, C25, and C36) were proved to be beneficial. Phase III: the identified beneficial positions and substitutions were analyzed by CompassR to find compatible substitutions that can be recombined in a beneficial manner. Phase IV: based on CompassR analysis the positions of S19, K10, N13 and K16 were recombined by SDM, yielding the variants V1 (S19K/K10L), V2 (S19K/K10L/N13H), and V3 (S19K/K10L/N13H/K16I). SSM: site‐saturation mutagenesis; CompassR: computer‐assisted Recombination; SDM: site‐directed mutagenesis.

The developed PLABS screening procedure was validated in a whole KnowVolution campaign comprising four phases (Phase I: identification, Phase II: determination, Phase III: selection, and Phase IV: recombination; see **Figure** **4**).

**Figure 5:**
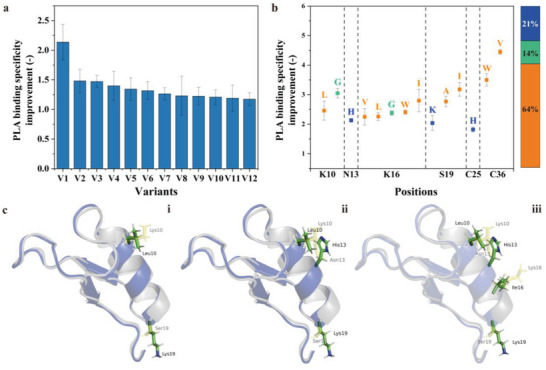
a) Variants with improved PLA binding specificity over PP obtained from Cg‐Def cepPCR library screening in Phase I. A value of 1 corresponds to the same PLA binding specificity over PP in Cg‐Def variants and WT. All experiments were performed in triplicate. b) Amino acid substitutions in variants with improved PLA binding specificity over PP in Phase II. Yellow, blue, and green indicates hydrophobic, positively charged, and neutral amino acids, respectively. All experiments were performed in triplicate. c) Models of three generated Cg‐Def variants including (i) V1, (ii) V2, and (iii) V3 generated with YASARA Structure Version 21.12.19.^[^ ^42^ ^]^ Graphical representations of models were generated using The PyMOL Molecular Graphics System, Version 2.0 Schrödinger, LLC. Beneficial positions (K10, N13, K16, and S19) are highlighted in green, and substitutions at each position are marked in yellow. Cg‐Def models were generated after energy minimization.

In Phase I, a Cg‐Def random mutagenesis library (cepPCR; 0.3 mM MnCl_2_) was generated and 2879 clones were screened toward increased PLA specific binding over PP. The mutation frequency was determined to be 23 mutations/kb, resulting in an average number of 2.5 amino acid substitutions per Cg‐Def variant. The screening results shows that 83.1% of Cg‐Def variants maintain its PLA binding preference (cut off: fluorescence ratio >0.8 times) compared to WT. The latter indicates a very good quality of cepPCR library. The “top 12 variants” with improved PLA binding preference over PP (**Figure** **5a**) were picked and sequenced (Table S4, Supporting Information). Sequencing resulted in 8 potential beneficial positions (K10, N13, K16, S19, C20, A22, C25, and C36). In Phase II, the 8 identified positions were subjected to site‐saturation mutagenesis (8 SSM libraries). After screening of 1408 clones, Cg‐Def variants with 1.5 times improved PLA binding specificity were identified and sent for sequencing (**Table** **1**). The top three variants (Cg‐Def C36V, Cg‐Def C36W, and Cg‐Def S19I) had a >3.0‐fold improved PLA binding specificity, which was mainly caused by the decreased PP binding (≈0.2 times of fluorescence of WT). Interestingly, at the positions of K10, N13, K16, S19, C25, and C36, substitutions to mainly hydrophobic or positively charged amino acids occurred (Figure 5b).

**Table 1:** Cg‐Def variants with improved PLA binding specificity over PP after site saturation of 8 positions and screening of 1408 clones^a)^.

Variants	PLA binding improvement	PP binding improvement	PLA binding specificity improvement from PP
Cg‐Def C36V	0.78 ± 0.04	0.19 ± 0.03	4.45 ± 0.08
Cg‐Def C36W	0.78 ± 0.01	0.22 ± 0.02	3.50 ± 0.13
Cg‐Def S19I	0.53 ± 0.03	0.17 ± 0.01	3.18 ± 0.23
Cg‐Def K10G	1.25 ± 0.27	0.74 ± 0.21	3.05 ± 0.16
Cg‐Def K16I	0.94 ± 0.2	0.33 ± 0.03	2.80 ± 0.38
Cg‐Def S19A	0.62 ± 0.03	0.22 ± 0.01	2.77 ± 0.18
Cg‐Def K10L	1.79 ± 0.31	0.71 ± 0.04	2.46 ± 0.32
Cg‐Def K16W	0.84 ± 0.03	0.35 ± 0.02	2.41 ± 0.07
Cg‐Def K16G	1.78 ± 0.06	0.78 ± 0.05	2.38 ± 0.08
Cg‐Def K16L	0.87 ± 0.07	0.38 ± 0.01	2.26 ± 0.13
Cg‐Def K16V	0.81 ± 0.06	0.37 ± 0.03	2.25 ± 0.28
Cg‐Def N13H	1.79 ± 0.51	0.85 ± 0.05	2.13 ± 0.06
Cg‐Def S19K	0.73 ± 0.05	0.37 ± 0.05	2.04 ± 0.25
Cg‐Def C25H	1.69 ± 0.06	0.96 ± 0.04	1.82 ± 0.03

^a)^ The experiments were performed in triplicate.

In Phase III, a computational CompassR analysis^[^ ^38^, ^39^, ^40^ ^]^ was performed to identify compatible amino acid substitutions that can be recombined to maximize beneficial improvements. CompassR analysis identified the substitutions K10L, N13H, K16I, and S19K as the most beneficial substitutions at the targeted positions (Figure S5, Supporting Information). Visualization of the 3D structure of Cg‐Def showed that identified positions are mainly located in the *α*‐helix of Cg‐Def.^[^ ^41^ ^]^ The top three ranked Cg‐Def recombinants are (Cg‐Def S19K/K10L (V1), Cg‐Def S19K/K10L/N13H (V2), and Cg‐Def S19K/K10L/N13H/K16I (V3)). These three variants were expressed, purified, and characterized in Phase IV (Figure 5c).

### 2.4 Characterization of Generated Cg‐Def Variants

#### 2.4.1 PLA Binding Specificity Characterization

**Figure 6:**
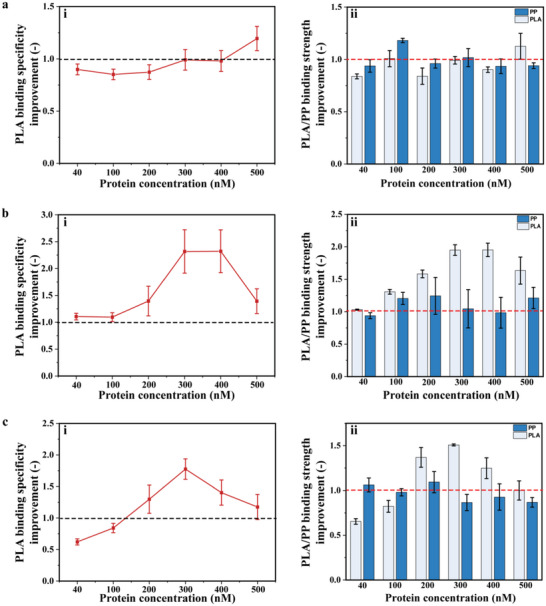
PLA binding specificity (i) and PLA/PP binding improvement (ii) of Cg‐Def variants including a) V1, b) V2, and c) V3. A value of 1 corresponds to the same PLA binding specificity over PP in Cg‐Def variants and WT. All experiments were performed in triplicate.

PLA binding properties of variants V1, V2, and V3 were characterized in PLA/PP MTPs with concentration ranging from 40 to 500 nM (**Figure** **6**). For V1 (Figure 6a), there was no significant change in the PLA binding specificity and PLA/PP binding improvement. While regarding to V2, PLA binding specificity reached highest improvement at Cg‐Def concentrations of 300 and 400 nM (2.3‐fold, Figure 6bi). V3 (Figure 6ci) showed a similar binding preference to V2 (1.8‐fold; at 300 nM). Changes in binding preference can be attributed to the improved PLA binding and/or reduced PP binding (Figure 6cii). V2 was finally selected for detailed biophysical characterizations due to its higher PLA binding specificity.

A final proof to quantify the improved binding preference was performed in a competitive binding assay in 2 mL glass vials containing punched PLA and PP discs as one pot experiment. **Table** **2** summarizes the change in the PLA/PP binding ratios of purified V2. The PLA/PP binding ratio increased from 0.53 (WT) to 0.85 (V2), indicating a significantly enhanced preference of PLA binding over PP.

**Table 2:** PLA binding specificity of Cg‐Def WT and V2 in measured fluorescence values and obtained ratio^a)^.

Variants	△PLA binding fluorescence value^b)^	△PP binding fluorescence value^c)^	△PLA:PP ratio^d)^
Cg‐Def WT	1834 ± 240	3489 ± 436	0.53 ± 0.02
V2	3352 ± 809	3932 ± 178	0.85 ± 0.19

^a)^ The fluorescence values of PLA and PP‐films were 909 and 1019 relative fluorescence units;

^b^ ^)^Subtracting fluorescence of EGFP bound PLA film from fluorescence of Cg‐Def WT/V2 bound PLA film to obtain △PLA binding fluorescence value;

^c^ ^)^Subtracting fluorescence of EGFP bound PP film from fluorescence of Cg‐Def WT/V2 bound PP film or to obtain △PP binding fluorescence value;

^d^ ^)^The △PLA:△PP ratio was calculated by dividing △PP binding fluorescence value by △PLA binding fluorescence value. The concentrations of Cg‐Def WT and V2 were 300 nM. All experiments were performed in triplicate.

All atomistic MD simulations of Cg‐Def WT and V2 on PLA and PP were performed using the GROMACS software. Therefore, contact frequencies and binding poses for WT and V2 on PLA and PP were derived. It was shown that V2 has a significantly altered final binding pose on the PLA surface compared to Cg‐Def WT (Figure S6, Supporting Information). The V2 bound state enables more residues, especially the substituted amino acids Leu10, His13, and Lys19 in sidechains of the *α*‐helix, to interact with PLA surface. We hypothesize that this increase in interacting residues translates into an increase in contributing forces, ultimately enhancing binding strength of V2 on PLA. Cg‐Def WT on PP and PLA and V2 on PP showed similar binding poses, all contacting the respective surface via the Leu30–Leu32 loop and the Pro5 turn region of Cg‐Def. It is visible that V2 has a slight increase in the total number of contact residues, explaining the minor improvement of V2 on PP binding.

#### 2.4.2 Surface Hydrophilicity

**Figure 7:**
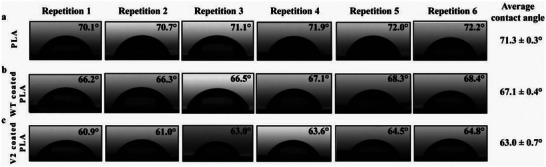
Contact angle measurements of distilled water on a) PLA‐film, b) PLA with Cg‐Def WT, and c) PLA with V2. The experiments were performed in six replicates.

Hydrophilicity of a PLA film and Cg‐Def WT/V2 coated PLA films was determined by simple contact angle measurements (**Figure** **7**). In case of a PLA film, a contact angle value of 71.3 ± 0.3° was measured (Figure 7a). PLA films coated with V2 yielded lower contact angles (63.0 ± 0.7°) compared to Cg‐Def WT (67.1 ± 0.4°), which demonstrated an increased hydrophilicity after V2 coating (Figure 7b and c).

#### 2.4.3 Surface Coverage by SPR

**Figure 8:**
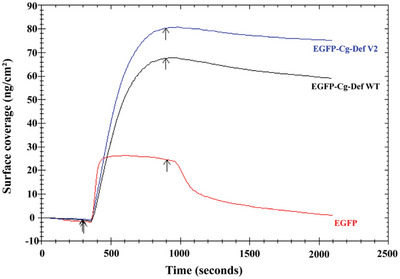
Comparison of surface coverages of EGFP, EGFP‐Cg‐Def WT, and EGFP‐Cg‐Def V2 on PLA coated SPR chips. Black arrows indicated the start and the end time of sample injection.

Surface coverage of Cg‐Def onto PLA was determined by SPR at saturated concentration of 500 nM for Cg‐Def WT, Cg‐Def V2 and EGFP (**Figure** **8**). Cg‐Def WT and V2 exhibited significantly enhanced surface coverage on PLA than EGFP (1.41 ng cm^−2^). V2 displayed a 1.30‐fold improved PLA surface coverage (75.36 ng cm^−2^) compared to Cg‐Def WT (59.50 ng cm^−2^) after 2000 s.

In essence, the reported screening platform provides a blueprint for engineering of material‐specific binding peptides since MBPs have been reported for various polymers,^[^ ^9^, ^11^, ^13^, ^19^ ^]^ metals and metal alloys,^[^ ^13^, ^14^ ^]^ and natural surfaces such as plant leaves.^[^ ^15^, ^43^ ^]^ Challenges comprise a high surface coverage (>60%) and an incubation time ranging from a few minutes up to 20 min. The fastest coating of MBP on carbon nanotubes with >90% coverage within 20 s was reported.^[^ ^44^ ^]^ However, no report exists in which a directed evolution campaign was performed to achieve high surface coverage with a reduced incubation time. Furthermore, it is unclear which protein fold can be evolved by protein engineering to achieve an antibody‐like material specificity.

## 3 Conclusion

In summary, a first PLABS high‐throughput screening platform to design and evaluate the protein engineering of material‐specific binding peptides has been developed and validated through a KnowVolution campaign. As a result, a Cg‐Def variant V2 with significantly improved PLA binding specificity over PP was yielded and SPR quantified the increased surface coverage (from 59.50 to 75.36 ng cm^−2^). The employed simple coating procedure for PLA on PP MTPs can very likely be expanded to other polymers such as PHA, PS, and ABS that dissolve in dichloromethane. The PLABS screening platform will enable in the future to design material‐specific binding peptides for polymers that can be crucial for a circular polymer economy through material‐specific labeling of polymer materials and targeted depolymerization of mixed plastic waste.

## 4 Experimental Section

All the methods are described in the Supporting Information.

## Conflict of Interest

The authors declare no conflict of interest.

## Author Contributions

U.S. provided the conceptual idea and work outline. Y.L., Y.J., and U.S. designed the experiments and competitive binding assay as final prove. Y.L. performed experiments and analyzed the data; Y.L. wrote the manuscript; K.‐W.H. did the computational analysis; Y.J., T.K., and U.S. revised the manuscript. All authors have given approval to the final version of the manuscript.

## Supporting information

Supporting Information

## Data Availability

The data that support the findings of this study are available from the corresponding author upon reasonable request.
