# Supplementary material for: A Competitive High‐Throughput Screening Platform for Designing Polylactic Acid‐Specific Binding Peptides
Source: Adv Sci (Weinh). 2023 Aug 23;10(29):2303195. doi: 10.1002/advs.202303195 (PMC10582454; doi:10.1002/advs.202303195)
Supplement: Supplementary file 1 — Supporting Information [file ADVS-10-2303195-s001.pdf]

ADVANCED SCIENCE
Open Access

Supporting Information

for *Adv. Sci.*, DOI 10.1002/advs.202303195

A Competitive High-Throughput Screening Platform for Designing Polylactic Acid-Specific Binding Peptides

Yi Lu, Kai-Wolfgang Hintzen, Tetiana Kurkina, Yu Ji and Ulrich Schwaneberg**

Supporting Information

A competitive high-throughput screening platform for designing polylactic acid-specific binding peptides

Yi Lu[1], Kai-Wolfgang Hintzen[1,2], Tetiana Kurkina[1], Yu Ji[1*]. Ulrich Schwaneberg[1,2*]

[1] Institute of Biotechnology, RWTH Aachen University, Aachen 52074, Germany

[2] DWI-Leibniz Institute for Interactive Materials, Aachen 52074, Germany

* Corresponding author e-mail: yu.ji@biotec.rwth-aachen.de; u.schwaneberg@biotec.rwth-aachen.de

1. Materials and Methods

1.1 Materials

All used chemicals were purchased from Sigma-Aldrich Corp. (St. Louis, USA), AppliChem GmbH (Darmstadt, Germany) as well as Carl Roth GmbH (Karlsruhe, Germany) and had analytical-reagent grade or higher purity. Synthetic genes were obtained from GeneArt AG (Regensburg, Germany) and oligonucleotides were acquired from Eurofins Scientific SE (Ebersberg, Germany) in salt-free form. Plasmid extraction and PCR purification kits were purchased from Macherey-Nagel GmbH & Co. KG (Düren, Germany). The BCA Protein Assay kit was obtained from Thermo Fisher Scientific Inc. (Rockford, USA). PLA (poly-L-lactic acid) granules (product code: ME34-GL-000180) with size of 3–5 mm were bought from Goodfellow GmbH (Huntingdon, England). PLA film (product code: ME33105011, thickness: 0.05 mm) and PP film (product code: LS526389, thickness: 0.50 mm) were bought from Goodfellow GmbH (Hamburg, Germany).

The plasmid pET28a(+) from Novagen (Darmstadt, Germany) was used as expression vector. *E. coli* strains DH5a and BL21-Gold (DE3) were purchased from Agilent Technologies Inc. (Santa Clara, USA). *E. coli* DH5a was used as cloning host and *E. coli* BL21-Gold (DE3) was used as protein expression system. MBPs binding was visualized by fluorescence of the fused reporter protein EGFP. All data represent as mean ± standard error.

1.2 Methods

1.2.1 Random mutagenesis library generation

Random mutagenesis was performed using cepPCR with modifications [1-2]. The Cg-Def gene (30 ng template pET28a(+)::EGFP-17xHelix-TEV-Cg-Def) was amplified with Taq DNA polymerase (1.25 U), mixed with deoxyribonucleotide triphosphates (dNTPs, 10 mM), Mn^{2+} (0.3 mM) and DNA primers F-cepCg-Def/R-cepCg-Def (20 μM each; see **Table S1**). The cepPCR conditions were: (95 °C for 2 min; one cycle), (95 °C, 30 s/59.9 °C, 30 s/72 °C, 20 s; 30 cycles) followed by a final step (72 °C, 10 min; one cycle). Parental DNA was digested (20 U DpnI, 16 h, 37 °C) and subsequently purified (PCR clean-up gel extraction kit). The amplification of the vector backbones pET28a(+)::EGFP-17xHelix-TEV-Cg-Def was performed using "megaprimer PCR of whole plasmid" (MEGAWHOP) [3]. The previously generated cepPCR products (35 ng) were used as megaprimers for amplification of templates with PCRBIO VeriFi™ polymerase (London, UK). The PCR conditions were: (72 °C, 3 min/95 °C, 1 min; one cycle), (98 °C, 15 s/62.9 °C, 1 min/72 °C, 3 min 6 s; 25 cycles) followed by a final elongation step (72 °C, 8 min; one cycle). The parental DNA was digested (20 U DpnI, 16 h, 37 °C), purified (PCR clean-up kit), and subsequently transformed into *E. coli* BL21-Gold (DE3) for expression.

1.2.2 Site-saturation mutagenesis and site-directed mutagenesis

Site-saturation mutagenesis (SSM) was performed at 8 positions of Cg-Def, including K10, N13, K16, S19, C20, A22, C25, and C36. The Cg-Def gene (5 ng template pET28a(+)::EGFP-17xHelix-TEV-Cg-Def) was amplified with PCRBIO VeriFi™ Mix (London, UK) and DNA primers (5 μM each; see **Table S2**). The SSM conditions were: (95 °C for 1 min; one cycle), (95 °C, 15s/50−70 °C, 15 s/72 °C, 3 min 6 s; 30 cycles). Parental DNA was digested (20 U DpnI, 16 h, 37 °C), purified (PCR clean-up gel extraction kit), and subsequently transformed into *E. coli* BL21-Gold (DE3) for expression.

Site-directed mutagenesis (SDM) was performed to generate Cg-Def variants (V1, V2, and V3) with improved PLA binding specificity. The genes Cg-Def (5 ng template pET28a(+)::EGFP-17xHelix-TEV-Cg-Def) were amplified with PCRBIO VeriFi™ Mix (London, UK) and DNA primers (5 μM each; see **Table S2**).The SDM conditions were same as that of SSM.

1.2.3 Expression of EGFP-Cg-Def in 96-well microtiter plates

Each transformant was transferred into one well of a 96-well MTP (flat-bottom, transparent, PS). The generation of glycerol stocks and the cultivation procedure were performed as

previously described [4]. Cell pellets were stored at -20 °C until use. *E. coli* BL21-Gold (DE3) cells harboring the plasmids of interest were resuspended in lysozyme solution (150 µL; 1.5 mg/mL, in 50 mM Tris-HCl buffer, pH 8.0) and incubated (1 h, 37 °C, 900 rpm, 70% humidity; Multitron Pro, Infors AG, Bottmingen, Switzerland), followed by centrifugation (3220 g, 30 min, 4 °C; Eppendorf centrifuge 5810 R, Eppendorf AG, Hamburg, Germany). Obtained supernatants were used directly for screening.

1.2.4 Expression of EGFP-Cg-Def in flasks for purification

The cultivation procedure for flask expression was performed as previously described [4]. The EGFP-MBP fusion proteins containing a N-terminal His_6-Tag were purified using a prepacked Ni-IDA 2000 column from Macherey-Nagel GmbH & Co. KG (Düren, Germany). Samples were desalted using an Amicon Ultra centrifugal tube with pore size of 10 kDa MWCO (Merck KGaA, Darmstadt, Germany).

1.2.5 Selection of MBP for PLA binding specificity

During selection of MBP for PLA binding specificity, purified EGFP-MBP proteins (See amino acid sequences of EGFP-MBPs in **Table S3**) were used to bind to PLA granules and PP MTPs. For binding with PLA granules, the binding test was performed in glass vials containing 200 µL EGFP-MBP solution (2.5 µM, in Tris-HCl, 50 mM, pH 8.0) as well as one PLA granule. After incubation for 10 min, PLA granules were washed with 200 µL Tris-HCl buffer (50 mM, pH 8.0) two times and 200 µL 0.1 mM surfactant alkyl benzene sulfonate (LAS). The binding experiments were repeated three times. Afterwards, the granules were analyzed by the fluorescence microscope (BX51, Olympus). The gain was set to 2 dB, and the exposure time tuned according to the background fluorescence of different granules. Untreated granules and EGFP bound granules were served as negative controls. Regarding to PP MTPs binding, 100 µL EGFP-MBP solution (2.5 µM, in Tris-HCl, 50 mM, pH 8.0) was transferred into wells of PP MTPs. After incubation for 10 min, PP MTPs were washed with 100 µL Tris-HCl buffer (50 mM, pH 8.0) two times and 100 µL 0.1 mM LAS. Residual fluorescence of the bound proteins was measured directly with the 96-well MTP reader CLARIOstar (exc. 485 nm, em. 520 nm, gain 1000, 35 reads/well).

1.2.6 Preparation of PLA coated MTPs

For preparing PLA coated MTPs, PLA granules were dissolved in dichloromethane with the PLA concentration in the range of 0.34–85 mg/mL. 50 µL of dissolved PLA solution was added

into PP MTPs and dried under 50 °C for 3 min. The PLA coated MTPs are ready for use when the dichloromethane was completely evaporated and PLA film was formed and attached firmly on the surface of PP MTP wells.

1.2.7 Screening of EGFP-Cg-Def library for improved PLA binding specificity

The screening of EGFP-Cg-Def library was conducted in PLA coated MTPs and PP MTPs. During screening process, 20 μL cell lysate of EGFP-Cg-Def was supplemented to 80 μL Tris-HCl buffer (pH 8.0, 50 mM) in PLA/PP MTP and incubated (10 min, room temperature, 600 rpm; MTP shaker, TiMix5, Edmund Bühler GmbH, Hechingen, Germany). In a subsequent washing step, the MTP wells were washed three times with Tris-HCl buffer (100 μL; pH 8.0, 50 mM, 5 min, room temperature, 600 rpm). In the final desorption step, 100 μL of LAS (pH 8.0, 0.5 mM) and Tris-HCl were supplemented to the wells and incubated (5 min, room temperature, 600 rpm) sequentially. After removal of excess liquid, the residual fluorescence of the bound EGFP-Cg-Def was measured directly on the well surface with the 96-well MTP reader CLARIOstar (exc. 485 nm, em. 520 nm, gain 1000, 35 reads/well). The improvement of PLA binding specificity was calculated by following the equation (1).

$$\text{Improvement of PLA binding specificity} = \frac{FI\ of\ V(PLA)/FI\ of\ WT\ (PLA)}{FI\ of\ V\ (PP)/FI\ of\ WT\ (PP)} \qquad (1)$$

Where FI represents fluorescence intensity; V represents variant; WT presents wide type. The value of improvement of PLA binding specificity is in the range of 0–5, and a change of 0.1 in the ratio indicates the significant fluctuation in the PLA or PP binding.

1.2.8 Relative folding free energies ($\Delta\Delta G_{fold}$) analysis for recombination of beneficial substitutions

The Computer-assisted Recombination (CompassR) strategy [5] was used to guide the recombination of beneficial substitutions. The relative folding free energies ($\Delta\Delta G_{fold}=\Delta G_{fold,sub}-\Delta G_{fold,wt}$) were computed using FoldX version 3b5.1 employing the YASARA Plugin in YASARA Structure version 19.12.4. The initial structure of the Cg-Def for analysis was taken from the Cg-Def crystal structure (PDB ID: 2B68). Default FoldX parameters were used for temperature 298 K, ionic strength 0.05 M, and pH 7. The structure of the Cg-Def WT was rotamerized and energy minimized using the "RepairObject" command to correct the residues that have non-standard torsion angles. Five FoldX runs were performed for each substitution to ensure that the minimum energy conformation of even large residues that possess many rotamers is identified. PyMOL was used to visualize the Cg-Def structure.

1.2.9 Characterization of PLA binding specificity

The recombined variants V1, V2, and V3 were purified and their PLA binding specificity was conducted in PLA/PP MTPs. During binding process, 100 µL purified variants with different concentrations (40–500 nM) was transferred in PLA/PP MTP and incubated (10 min, room temperature, 600 rpm). In a subsequent washing step, the MTP wells were washed three times with Tris-HCl buffer (100 µL; pH 8.0, 50 mM, 5 min, room temperature, 600 rpm). In the final desorption step, 100 µL of LAS (0.5 mM) and 100 µL of Tris-HCl were supplemented to the wells and incubated (5 min, room temperature, 600 rpm) sequentially. After removal of excess liquid, the residual fluorescence of the bound variants was measured directly on the well surface with the 96-well MTP reader CLARIOstar (exc. 485 nm, em. 520 nm, gain 1000, 35 reads/well).

In order to determine the PLA binding specificity of variants in competitive binding to different polymers in one pot, PLA and PP films were punched by press machine (Gechter 12 kN HKPL-DS, Obermichelbach, Germany) into identical discs, which have same size to the well of MTPs (diameter of 6.39 mm). During binding process, PLA and PP discs were put into glass vials (E159.1, Carl Roth, Karlsruhe, Germany) containing 2 mL purified EGFP, EGFP-Cg-Def WT, and EGFP-V2 solutions with certain concentration (300 nM, in Tris-HCl) and incubated (10 min, room temperature, rotate speed: 20 rpm/min). In a subsequent washing step, the films were washed three times with Tris-HCl buffer (2 mL; pH 8.0, 50 mM, 5 min, room temperature, rotate speed: 20 rpm/min). In the final desorption step, 2 mL of LAS (0.5 mM) and 2 mL of Tris-HCl were supplemented into the vials with the films inside and incubated (5 min, room temperature, rotation speed: 20 rpm/min). After removal of excess liquid, the films were put into wells of black PP MTPs and the residual fluorescence of the bound proteins was measured directly with the 96-well MTP reader CLARIOstar (exc. 485 nm, em. 520 nm, gain 1000, 35 reads/well).

1.2.10 Determination of water contact angle

The determination of static contact angles (sessile drop) was performed by the Drop Shape Analyzer DSA100S (Krüss GmbH, Hamburg, Germany). For the sample preparation, PLA and PP films (discs with diameter of 6.39 mm) were incubated with 2 mL EGFP, EGFP-Cg-Def WT, and EGFP-V2 solutions (500 nM; in Tris-HCl, 50 mM, pH 8.0). After incubation for 10 min, PLA and PP films were washed with 2 mL Tris-HCl buffer (50 mM, pH 8.0) and dried with nitrogen. Contact angle was measured in distilled water at room temperature. A constant volume of water (3 µL) was deposited with dosing speed of 250 µL/min on the surface of

prepared PLA and PP films and the contact angle value was calculated from the recorded droplet image.

1.2.11 Determination of binding affinity to PLA

The binding affinity of EGFP, EGFP-Cg-Def WT, and EGFP-Cg-Def V2 on PLA coated chips were assessed by surface plasmon resonance (SPR) spectroscopy with concentration of 500 nM using a MP-SPR Navi™ 420A ILIVES four-channel SPR system (BioNavis Ltd, Tampere, Finland) at 785 nm. To prepare PLA coated SPR chip, 1% PLA solution (in chloroform) was prepared and 100 µL of PLA solution was put into the surface of pure gold chip for spinning coating (2000 rpm, 30 s; MODEL WS-650SZ-6NPP/LITE).

Protein solutions in Tris-HCl buffer (50 mM, pH 8.0) was flown over the PLA coated SPR chip with the speed of 20 µL/min for 10 min while the position of the plasmons was recorded. After the adsorption reached a plateau, Tris-HCl buffer (50 mM, pH 8.0) was introduced with the speed of 20 µL/min for 20 min. The amount of adsorbed protein was extracted from the sensor response in µRIU and was calculated as the difference between the baseline in buffer before and after protein injection (ΔµRIU) and converted into surface coverage (Q) in ng/cm^2 using SPR-Navi Data Viewer (Version 6.4.0.7).

Figures

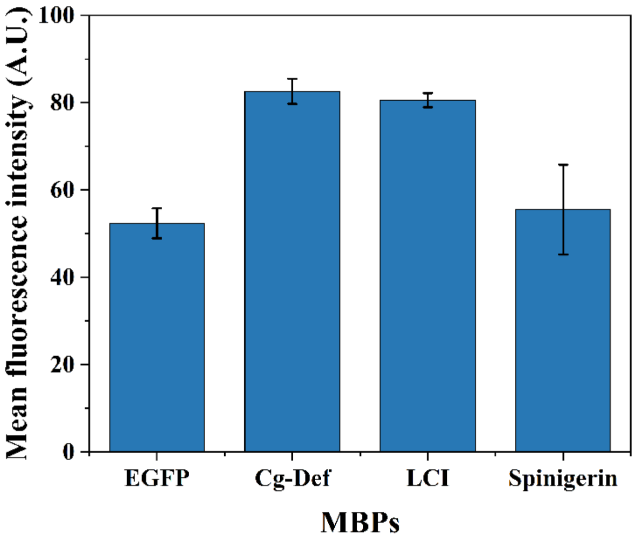

Figure S1. Comparison of PLA binding performance of EGFP and three MBPs including Cg-Def, LCI and Spinigerin. PLA binding of EGFP and three MBPs was indicated by mean fluorescence intensity (in arbitrary units, A.U.), which was measured based on fluorescence microscopy images of MBPs bound PLA granules using ImageJ software. The experiments were performed in triplicate.

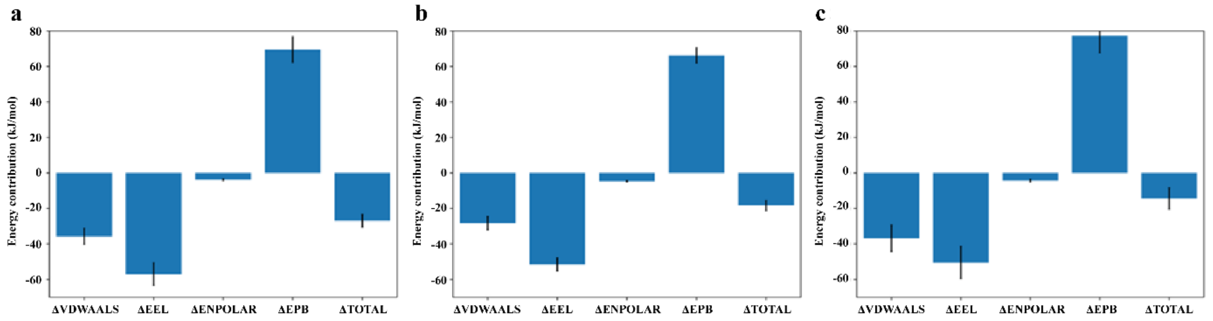

Figure S2. Binding free energies according to Molecular Mechanics/Poisson-Boltzman Surface Area (MM/PBSA) calculations executed in gmx_MMPBSA6 for (a) Cg-Def on PLA, (b) LCI on PLA, and (c) Spinigerin on PLA. ΔVDWAALS, ΔEEL, ΔENPOLAR, and ΔEPB represent the van der Waals energy contributions, electrostatic contributions, non-polar contributions, and polar contributions to the total binding free energy (ΔTOTAL), respectively.

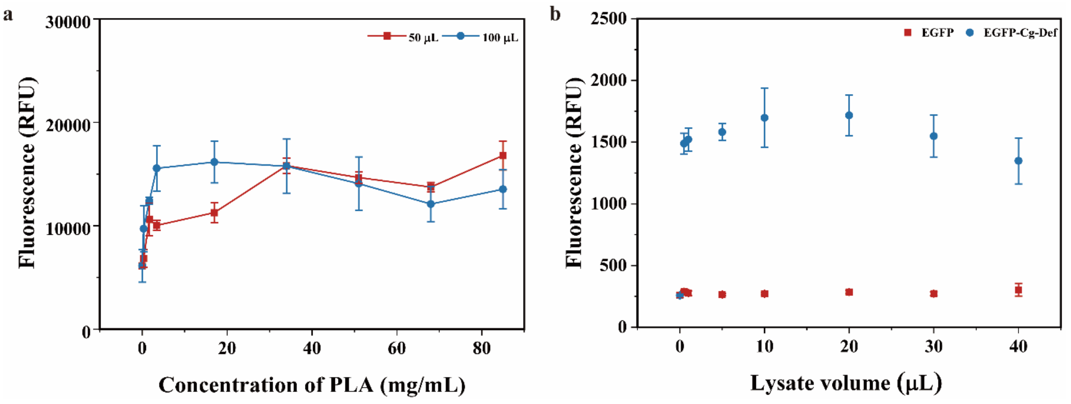

Figure S3. Optimization of PLA coating concentration (a) and lysate volume (b) for screening system. An optimized coating process was developed employing 50 μL of PLA solution (in dichloromethane, 34 mg/mL). The concentration of Cg-Def was 1 μM for PLA coating concentration optimization. The lysate volume of EGFP and EGFP-Cg-Def in the range of 0–40 μL were analyzed and the saturated binding onto PLA MTPs was achieved by applying 20 μL of EGFP-Cg-Def.

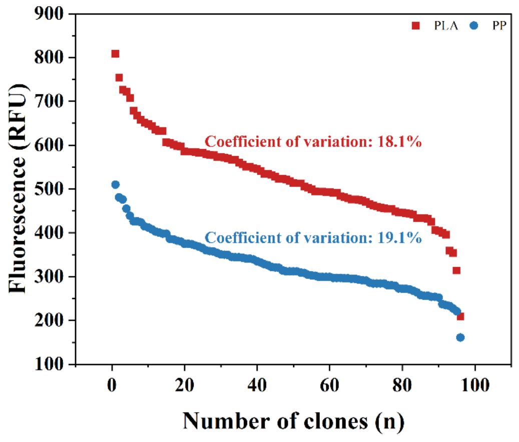

Figure S4. The coefficient of variation of screening system in PLA coated and PP MTPs. Detected fluorescence in PLA coated and PP MTPs incubated with EGFP-Cg-Def was in descending order.

	K10	N13	K16	S19	C25	C36
**V**	0.735263	1.23937	1.51624	0.296937	6.33427	4.25745
**Y**	0.265098	-0.12462	1.74502	-0.20512	9.51815	2.46234
**W**	0.042197	0.409198	1.83772	-0.46776	13.9576	2.58221
**T**	0.928264	1.99531	1.95933	0.558326	5.30351	5.77203
**S**	0.759074	0.307595	1.16561	1.61E-11	5.17E+00	4.28E+00
**P**	-1.20591	3.42462	3.53265	1.94906	4.3759	3.18402
**F**	0.0891069	0.179908	1.5519	-0.17864	6.43234	2.29397
**M**	-0.491837	-0.75794	0.545369	-0.71567	4.98343	2.24474
**K**	-0.0231871	-0.34114	-0.00424	-0.55188	5.73921	2.77486
**L**	-0.477093	-0.79868	1.23088	-0.29365	6.50273	2.23129
**I**	0.121367	0.368174	0.891418	0.09674	7.21996	6.3635
**H**	0.603743	0.755325	1.44607	0.047163	10.744	3.7558
**G**	1.02077	1.33199	1.75293	-2.26601	3.96376	4.33066
**E**	-0.0188196	-0.14432	1.05973	-0.26993	6.51152	3.50384
**Q**	3.45E-01	-3.93E-02	6.20E-01	-1.99E-01	6.33E+00	3.87E+00
**C**	7.96E-01	7.81E-01	1.14E+00	-4.06E-01	-1.18E-08	1.70E-02
**D**	0.538534	1.19821	2.13035	-0.07727	6.85747	5.0864
**N**	-0.242927	-0.00093	1.19011	-0.06072	6.42155	3.88188
**R**	0.399869	0.537363	-0.6379	-0.14504	5.77067	2.86339
**A**	0.33885	0.38464	1.19807	-0.00924	4.19402	3.47446

Destabilizing **Neutral** **Stabilizing**

Figure S5. $\Delta\Delta G_{fold}$ of substitutions in the positions of K10, N13, K16, S19, C25, and C36 of Cg-Def. Green and red colors indicated the low and high $\Delta\Delta G_{fold}$ value, respectively.

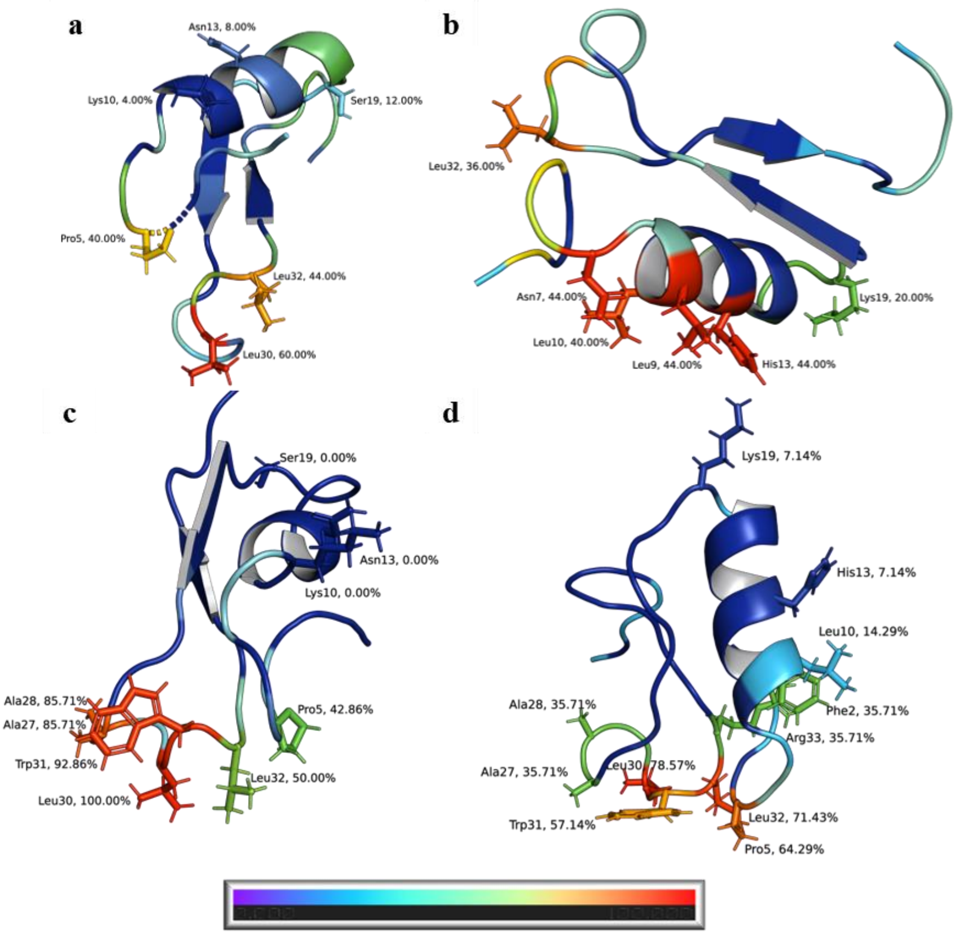

Figure S6. Contact frequencies of (a) Cg-Def WT on PLA, (b) V2 on PLA, (c) Cg-Def WT on PP, and (d) V2 on PP. Contact frequencies are color-coded onto the most common binding pose of the respective peptide on the polymer surface.

Tables

Table S1. Sequences of primers used in cepPCR.

Primer name	Sequence 5'→ 3'
F-cepCg-Def	GCAGAAAATCTGTATTTTCAGGGT
R-cepCg-Def	CAGCCGGATCTCAGTGTTATTA

Table S2. Sequences of primers used in SSM and SDM.

PCR	Primer name	Sequence 5' → 3'
SSM	F-K10X	CAGCTGNNKTGTAACAATCATTGCAAAAG
	R-K10X	TGATTGTTACAMNNCAGCTGATTACCCG
	F-N13X	TGTAACNNKCATTGCAAAAGCATTAGCTGC
	R-N13X	GCTTTTGCAATGMNNGTTACATTTCAGCTG
	F-K16X	AACAATCATTGCNNKAGCATTAGCTGCCGT
	R-K16X	ATGCTMNNGCAATGATTGTTACATTTCAGCTG
	F-S19X	GCATTNNKTGCCGTGCAGGTTATTGTG
	R-S19X	CTGCACGGCAMNNAATGCTTTTGCAATG
	F-C20X	GCATTAGCNNKCGTGCAGGTTATTG
	R-C20X	TGCACGMNNGCTAATGCTTTTGCAATG
	F-A22X	TAGCTGCCGTNNKGGTTATTGTGATGCAGC
	R-A22X	CAATAACCMNNACGGCAGCTAATGCTTTTG
	F-C25X	GGTTATNNKGATGCAGCAACCCTGTGGC
	R-C25X	GCTGCATCMNNATAACCTGCACGGC
	F-C36X	GCGTTGTACCNNKACCGATTGTAATGGC
	R-C36X	CAATCGGTMNNGGTACAACGCAGCCACAG
SDM	F-V1	GTAATCAGCTGCTGTGTAACAATCATTGCAAAAGCATTAAGTGCCGTG CAG
	R-V1	GATTGTTACACAGCAGCTGATTACCCGGACAACCAAAACC
	F-V2	GTAATCAGCTGCTGTGTAACCATCATTGCAAAAGCATTAAGTGCCGTG CAG
	R-V2	GCAATGATGGTTACACAGCAGCTGATTACCCGGACAACCAAAACC
	F-V3	GTAATCAGCTGCTGTGTAACCATCATTGCATTAGCATTAAGTGCCGTG CAG
	R-V3	GCAATGATGGTTACACAGCAGCTGATTACCCGGACAACCAAAACC

Table S3. Amino acid sequences of MBPs.

MBPs	Amino acid sequence (5' → 3')
Cg-Def	GFGCPGNQLKCNNHCKSISCRAGYCDAATLWLRCTCTDCNGKK
LCI	AIKLVQSPNGNFAASFVLDGTKWIFKSKYYDSSKGYWVGIYEVWDRK
Spinigerin	HVDKKVADKVLLLKQLRIMRLLTRL

Table S4. Amino acid substitutions in Cg-Def variants with high PLA binding specificity from PP screened in Phase I of KnowVolution.

Variants	Substitutions
V1	S19N
V2	K10I, C25W
V3	N13Y
V4	K16R, A22V
V5	C36S
V6	C20S, D38E
V7	F2L, C34R
V8	C20R, L32P
V9	L9P, L30Q, T37I
V10	S19R
V11	K10I, A22V
V12	C20Y

Reference

[1] R. C. Cadwell, G. F. Joyce, *PCR Methods Appl.* **1992**, *2* (1), 28.

[2] J. H. Yang, A. J. Ruff, M. Arlt, U. Schwaneberg, *Biotechnol. Bioeng.* **2017**, *114* (9), 1921.

[3] K. Miyazaki, in *Methods in Enzymology*, (Ed.: C. Voigt), Elsevier Academic Press Inc, San Diego **2011**, Ch. 17.

[4] K. Rubsam, B. Stomps, A. Boker, F. Jakob, U. Schwaneberg, *Polymer* **2017**, *116*, 124.

[5] H. Cui, H. Cao, H. Y. Cai, K. E. Jaeger, M. D. Davari, U. Schwaneberg, *Chemistry-a European Journal* **2020**, *26* (3), 643.
